# Developing a Reproducible Microbiome Data Analysis Pipeline Using the Amazon Web Services Cloud for a Cancer Research Group: Proof-of-Concept Study

**DOI:** 10.2196/14667

**Published:** 2019-11-11

**Authors:** Jinbing Bai, Ileen Jhaney, Jessica Wells

**Affiliations:** 1 Nell Hodgson Woodruff School of Nursing Emory University Atlanta, GA United States; 2 Cancer Prevention and Control Program Winship Cancer Institute Emory University Atlanta, GA United States; 3 Winship Research Informatics Shared Resource Winship Cancer Institute Emory University Atlanta, GA United States

**Keywords:** Amazon Web Services, cloud computation, microbiome, pipeline, sequence analysis

## Abstract

**Background:**

Cloud computing for microbiome data sets can significantly increase working efficiencies and expedite the translation of research findings into clinical practice. The Amazon Web Services (AWS) cloud provides an invaluable option for microbiome data storage, computation, and analysis.

**Objective:**

The goals of this study were to develop a microbiome data analysis pipeline by using AWS cloud and to conduct a proof-of-concept test for microbiome data storage, processing, and analysis.

**Methods:**

A multidisciplinary team was formed to develop and test a reproducible microbiome data analysis pipeline with multiple AWS cloud services that could be used for storage, computation, and data analysis. The microbiome data analysis pipeline developed in AWS was tested by using two data sets: 19 vaginal microbiome samples and 50 gut microbiome samples.

**Results:**

Using AWS features, we developed a microbiome data analysis pipeline that included Amazon Simple Storage Service for microbiome sequence storage, Linux Elastic Compute Cloud (EC2) instances (ie, servers) for data computation and analysis, and security keys to create and manage the use of encryption for the pipeline. Bioinformatics and statistical tools (ie, Quantitative Insights Into Microbial Ecology 2 and RStudio) were installed within the Linux EC2 instances to run microbiome statistical analysis. The microbiome data analysis pipeline was performed through command-line interfaces within the Linux operating system or in the Mac operating system. Using this new pipeline, we were able to successfully process and analyze 50 gut microbiome samples within 4 hours at a very low cost (a c4.4xlarge EC2 instance costs $0.80 per hour). Gut microbiome findings regarding diversity, taxonomy, and abundance analyses were easily shared within our research team.

**Conclusions:**

Building a microbiome data analysis pipeline with AWS cloud is feasible. This pipeline is highly reliable, computationally powerful, and cost effective. Our AWS-based microbiome analysis pipeline provides an efficient tool to conduct microbiome data analysis.

## Introduction

Big data and data-driven analysis has become a primary driver of precision health [1,2]. The human microbiota and their genomes, collectively called the human microbiome, is one form of big data [3]. The human body harbors trillions of microbes, including bacteria, viruses, fungi, and archaea [4,5], which vary from host to host and across body sites within a single host [6,7]. The human microbiome plays a critical role in human health and disease [8,9]. With advances in next-generation sequencing technology and the rise of shotgun metagenomics and metabolomic techniques, microbiome data sets have rapidly expanded, especially following the initiatives of the Human Microbiome Project [10] and the American Gut Project [11]. Computation and analysis of big data sets in local infrastructures via traditional computational methods (eg, use of personal computers and local computational clusters) often requires prolonged run times, delaying further analytic work that needs to be performed and postponing the translation of research findings into clinical practice [12]. Another shortcoming of classical data analysis methods is the difficulty involved in sharing the data and findings among research collaborators. Advances in cloud computing have provided the technical capabilities to help resolve difficulties posed by standard computational methods [12,13].

Beyond data storage, assessing human microbiome data sets requires bioinformatic tools that enable deeper mining, the deciphering of the mechanistic connections among the microbes, and the potential functions of these communities. To examine significant associations between metadata (eg, demographic and clinical variables) and DNA sequencing data, special bioinformatic and statistical tools for conducting microbiome analyses are needed [14]. It was not until recently that researchers have developed software for microbiome data analysis (eg, Quantitative Insights Into Microbial Ecology [QIIME] and Mothur) [15]. One popular bioinformatics tool, QIIME 2, can be installed natively within a conda environment through a docker or a cloud platform. The Amazon Web Services (AWS) cloud provides an invaluable computational environment for running bioinformatics tools, such as QIIME 2, without the overhead of implementing and supporting a large-scale computing infrastructure [12]. Cloud-based computational pipelines have been developed for a variety of data analysis, including CloudNeo [16] and RNA-Sequencing (RNA-Seq) Analysis Pipeline [17], for next-generation sequencing data, and Clustered Regularly Interspaced Short Palindromic Repeats Cloud (CRISPRcloud) for the deconvolution of pooled screening data [18]. The development of a comprehensive microbiome data analysis pipeline, including data storage, computations, and analysis, along with its testing using microbiome data sets from actual studies, would help researchers further investigate the impact of the microbiome on human health and disease (eg, cancer, metabolic syndromes, and neurodegenerative disorders) [8,19-21]. A reliable and validated microbiome data analysis pipeline operating through the AWS cloud could be used to provide a consistent communication platform for research collaborators to share information on data processing, data analysis, and research findings. Thus, the AWS pipeline could increase both the reproducibility of microbiome studies and the proficiency of the research team [22].

For clinical scientists, several challenges need to be overcome before conducting microbiome projects: (1) collective storage space for big microbiome data sets needs to be created so that the data and results can be easily shared within the research team; (2) centralized data computing capabilities need to be established to foster the replicability of results across all current and future projects; and (3) the cost for cloud computing services needs to be determined so that the team can cost-effectively study the human microbiome. The AWS cloud has become a popular platform for big data storage, high performance computing, and analytics [16-18,23]. Thus, the purpose of this study was to develop a microbiome data analysis pipeline using the AWS cloud service (MAP-AWS) and conduct a proof-of-concept test for microbiome data computation and analysis with this newly developed MAP-AWS.

## Methods

### Overview

**Figure 1:**
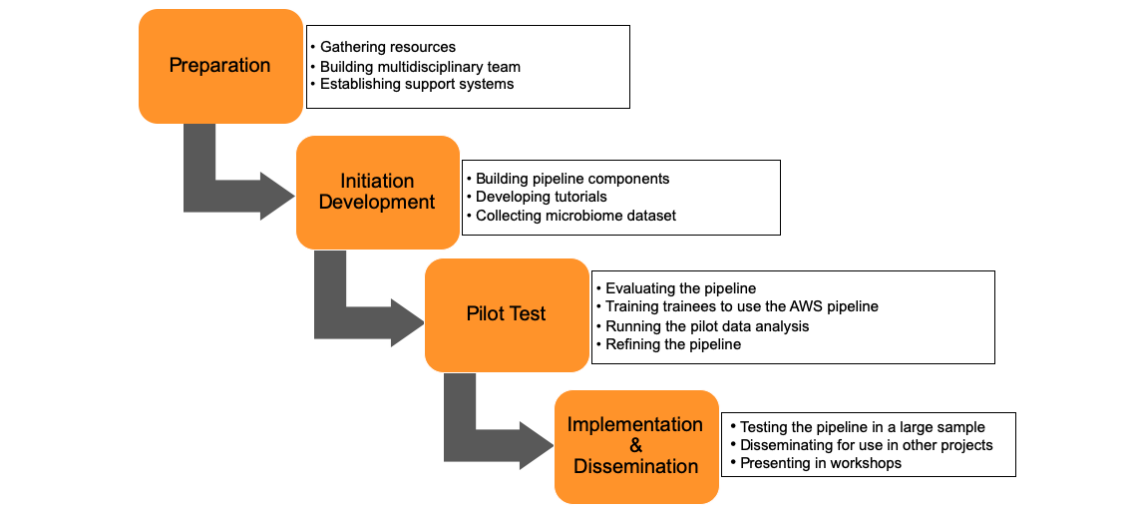
Design process of the microbiome analysis pipeline.

The process of developing and testing the MAP-AWS comprised of four stages, as illustrated in Figure 1. We first collected resources regarding microbiome data analysis and the use of MAP-AWS, built a multidisciplinary research team, and lined up available support systems from our institution and the AWS Support Center. Second, we initiated the development of the microbiome analysis pipeline, including a tutorial, with support of the Research Informatics Team from the Winship Cancer Institute at Emory University (Atlanta, Georgia, USA). 

Third, we began pilot testing: we ran a small vaginal microbiome data set (n=19), refined the pipeline and the tutorial, and retested the pipeline with a larger data set from the gut microbiome (n=50). Last, we disseminated the MAP-AWS within our institutional research groups via presentations and workshops and obtained feedback regarding this newly developed MAP-AWS.

### Preparation

Through our previous work analyzing microbiome data sets [22,24], we found that assembling a team with the necessary skill sets, ensuring financial feasibility, and establishing system resources were essential components of developing a system for big data analysis. Building a multidisciplinary team with specific expertise is key to successfully deploying AWS cloud for use in microbiome data processing, computing, and analysis. The MAP-AWS team was built within a multidisciplinary group of nurses, physicians, biostatisticians, epidemiologists, and microbiologists from the Schools of Nursing, Public Health, and Medicine, and the Winship Cancer Institute at Emory University. One nurse researcher, primarily trained in the human microbiome and cancer science, led the team and formed extensive collaborations with several other team members: one research informatics analyst from the Winship Cancer Institute, one biostatistician from the School of Public Health, and one predoctoral student from the School of Nursing. All team members had considerable experience in microbiome data analysis [24,25], including previous QIIME 2 training, active participation in microbiome-related internal and external workshops, and publications on the human microbiome and its impact on human health [24,25].

Recruiting information technology (IT) resources early in the preparation phase is critical to ensuring that all IT resources are coordinated by the time they are needed, and support services for troubleshooting activities can be provided in a timely manner. IT staff can help coordinate and organize the diverse data sets that will be used, including the DNA sequencing data, metadata, project-related information, and research protocols. Assignment of policies and permissions for access to all AWS resources is a critical role of the Research Informatics IT group. Assistance with AWS command-line interface (CLI) was provided through the IT support group as well. We set up AWS accounts for each team member intensively involved in developing the MAP-AWS with the support of our IT support group and the online AWS Support Center.

### Development of the Microbiome Data Analysis Pipeline Using Amazon Web Services

AWS cloud provides various options for microbiome data storage, computing, and analysis. For data storage, the Amazon Simple Storage Service (S3) buckets were used. For microbiome data computing, Amazon Elastic Compute Cloud (EC2) instances (ie, virtual servers in the AWS cloud) were primarily used, and for microbiome data analysis and specific bioinformatics, statistical packages that included QIIME 2 [22] and RStudio (RStudio, Boston, Massachusetts), were installed within the EC2 instances. For most of the EC2 instances we created we opted to use the Linux operating system, which has an optimized central processing unit (CPU), memory, and storage configurations [26]. During this specific developmental stage, we produced a step-by-step tutorial on how to run processes on microbiome data sets within AWS. This tutorial included the topics: logging into AWS, data importation and storage, data analysis using QIIME 2, and exportation of analysis results. This newly developed MAP-AWS provides a complete workflow to run microbiome data analysis in AWS.

**Figure 2:**
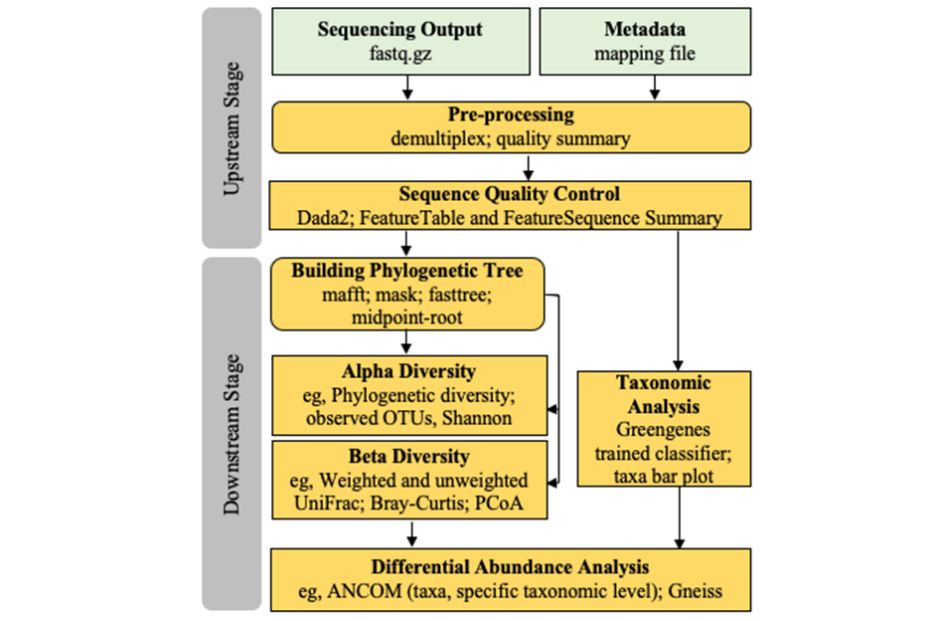
QIIME 2 workflow. QIIME: Quantitative Insights Into Microbial Ecology; OTU: operational taxonomic unit; PCoA: principal coordinates analysis; ANCOM: analysis of composition of microbiomes.

The use of QIIME 2 for microbiome data analysis has been tested by our group in a variety of computer systems, such as the Linux operating system, the Mac operating system (OS), and AWS cloud [24]. The QIIME 2 pipeline generates the bacterial community’s information for each sample [22], and this process includes two phases which are referred to as the upstream and downstream stages (Figure 2). The upstream stage consists of importing 16S rRNA sequences, ensuring sequence quality control, constructing a feature table, and generating a phylogenetic tree which illustrates the ecologic similarities of the bacterial taxa present in a sample [24]. The feature table describes the features present and the number of samples associated with each feature in the sample set. The downstream stage consists of taxonomic, diversity, and abundance analysis [14,22]. In this stage, statistics and interactive visualizations of the data are used to display the findings via figures and tables [27].

### Testing the Microbiome Data Analysis Pipeline Using Amazon Web Services

To test the feasibility of the MAP-AWS, we undertook three rounds of testing using a case-based formative approach to refine the microbiome analysis pipeline. In round one, we trained two novice students to use the MAP-AWS to determine where major changes were needed to improve the usability of the content and presentation formats of the tutorial and pipeline. Then, we demonstrated the MAP-AWS to a group of cancer scientists to get feedback regarding the content and presentation of the tutorial. In round two, we conducted a pilot test of the workflow in AWS cloud with a small training data set (19 deidentified vaginal microbiome samples from women with gynecologic cancers), which we had prepared for the purpose of training research scientists in microbiome data analysis [24]. This step enabled us to identify and troubleshoot issues before running a larger microbiome data set. In round three, two team members (JB and IJ) independently analyzed the same vaginal microbiome data set (ie, 16S rRNA V3-V4 gene sequences with corresponding metadata) using the MAP-AWS with the same Greengenes classifiers to determine the reproducibility of the pipeline. Final findings were compared between the two team members. Lastly, we ran a larger sample (50 deidentified gut microbiome samples) sequenced by the Emory Integrated Genomics Core. For each project, we regularly tracked costs and the processing times using the built-in QIIME 2 provenance feature that captures system environment variables, including processing time and system versions (ie, Linux and QIIME).

### Dissemination of the Microbiome Data Analysis Pipeline Using Amazon Web Services

After testing and refining the MAP-AWS processes and tutorial, we expanded the use of this pipeline to other microbiome projects within our team, including a gut microbiome and colorectal carcinogenesis study which involved sequence and metadata import, data quality control, results analysis, and model building. We disseminated our pipeline through presentations and workshops.

### Ethical Consideration

All the microbiome data we used in this study have been deidentified and no Institutional Review Board (IRB) approval is needed.

## Results

### Description of the Developed Microbiome Data Analysis Pipeline Using Amazon Web Services

**Figure 3:**
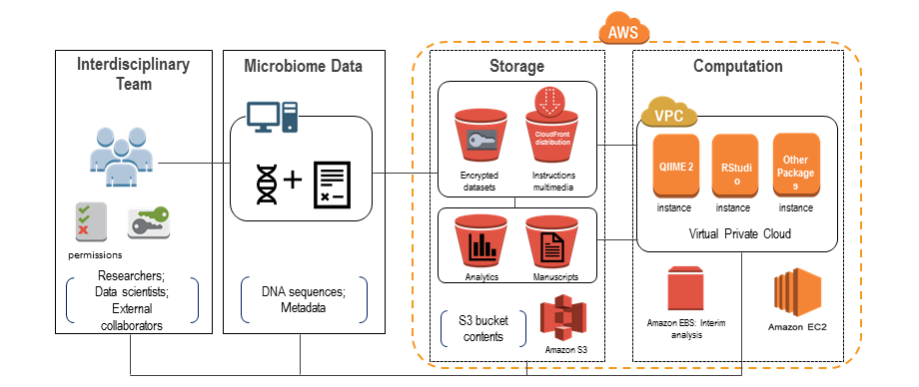
The microbiome data analysis pipeline using AWS. AWS: Amazon Web Services; S3: Simple Storage Service; VPC: virtual private cloud; QIIME: Quantitative Insights Into Microbial Ecology; EBS: Elastic Block Store; EC2: Elastic Compute Cloud.

The main components of the MAP-AWS include a multidisciplinary research team, bacterial sequences and corresponding metadata, Amazon S3 buckets for microbiome data storage, Linux EC2 instances (with QIIME 2 and RStudio installed) to run microbiome data analysis, and security keys to create and manage the use of encryption (Figure 3). With our platform, microbiome data analysis can be performed using AWS’s CLI within the Linux operating system or in the Mac OS system.

We primarily used S3 buckets for storage and EC2 instances for analysis. With our MAP-AWS, we used two specific types of storage. The first was Amazon Elastic Block Store (EBS), which is closely integrated with our EC2 instance. EBS is used to store hard drive contents of EC2 instances, as well as snapshots of these instances [28]. The other storage class used was Amazon S3 bucket, which is simply cloud object storage. We carefully planned permissions for storage, encryption, and EC2 instance access. The AWS EC2 provides virtual machines that are optimized for running CPU-intensive cloud-based applications [28]. Depending upon the analysis and virtual server purpose, our EC2 instances were configured for general purpose or optimized specifically for memory, computational power, or storage. For each EC2 instance, we were able to specify random access memory (RAM), virtual CPUs, storage, and network performance.

A Secure Shell (SSH) client (either a Mac OS terminal or MobaXterm for Windows) was used to securely connect to our EC2 instances, enabling remote access to a terminal through which Linux commands could be entered to process data. The AWS CLI was installed and used extensively to interact with our AWS resources and infrastructure.

Next, we set up encryption to ensure our S3 buckets were secured from unauthorized access. We assigned encryption at the bucket level so that all objects moved into the bucket were automatically encrypted.

### Testing of the Microbiome Data Analysis Pipeline Using Amazon Web Services

#### Feasibility

Two undergraduate nursing students were trained to use the MAP-AWS tutorial we developed and were interviewed after they finished performing analysis of the training microbiome data set (the vaginal microbiome samples). They both successfully completed the training data set analysis under the guidance of the tutorial and our team member (IJ). Both undergraduates were positive about the use of the MAP-AWS and the tutorial, supporting the feasibility of the MAP-AWS.

#### Reproducibility

All steps performed using the MAP-AWS in this study were tested with support from the QIIME 2 Development Team and the AWS Support Center. A total of three incidents involving the S3 bucket and EC2 instances needed addressing and were resolved by the AWS Support Center during the MAP-AWS development process. Two trained microbiome team members (JB and IJ) independently analyzed the same vaginal microbiome data set (ie, 16S rRNA V3-V4 gene sequences with the corresponding metadata, n=19) using the MAP-AWS. Comparisons of the final findings showed identical results in upstream and downstream analyses (Figure 2) [24], supporting the reproducibility of the MAP-AWS for microbiome data analysis.

#### Cost and Efficiency

The 50 gut microbiome samples were successfully processed within 4 hours with the MAP-AWS and subsequently processed for microbiome diversity, taxonomy, and abundance analyses using QIIME 2, version 2018.4 (Table 1). Performing microbiome data analysis for the same data set with typical client-server architecture took >6 hours. These running times were retrieved from the QIIME 2 provenance. We duplicated our efforts on a more recent QIIME 2, version 2019.4, and the running times for completion were congruent with previous results. Compared with standard methods of microbiome data analysis, the MAP-AWS processed these samples efficiently and at a low cost. We used a c4.4xlarge EC2 instance, which costs $0.80 per hour. This pricing level is similar to the Nephele pipeline published in 2017, a c3.4xlarge EC2 instance at the time costing $0.84 per hour [23].

**Table 1:** Running Time for the Gut Microbiome Sample Analysis (n=50).

Stage, step		MAP-AWS^a^		Traditional methods	
**Upstream stage**					
	Data import		4 s		1 min 48 s
	Quality control (ie, Dada2)		3 h 22 min 27 s		6 h 38 min 49 s
	Phylogeny		32 s		<1 min
**Downstream stage**					
	Taxonomy analysis		1 min 23 s		3 min 27 s
	Diversity analysis		2 s		8 s

^a^MAP-AWS: Microbiome Data Analysis Pipeline Using Amazon Web Services.

## Discussion

### Key Findings

This paper developed a microbiome data analysis pipeline by using AWS cloud and conducted a proof-of-concept test for microbiome data storage, processing, and analysis. This pipeline is highly reliable, computationally powerful, and cost effective. This study was a proof of concept for building and testing a newly developed pipeline (MAP-AWS) for microbiome data analysis. This pipeline is efficient and highly cost effective. It will provide a convenient environment to share analysis tools and results between collaborators. To accurately assess and utilize this data, we rely on the development of tools, pipelines, and standard operating procedures to handle big data effectively and efficiently via the AWS cloud. Microbiome pipelines using on-demand EC2 instances showed a great capacity for microbiome data analysis at a low cost. This pipeline improved productive and insightful collaboration with clinical scientists across different institutions to help the multidisciplinary research team continue the collaborative use of AWS.

With growing interest in evaluating the human microbiome and deciphering its relationship with health and disease, more efficient and cost-effective tools are needed for microbiome big data analysis. The purpose of this study was to develop and evaluate the MAP-AWS platform for use by clinical scientists. We described how researchers can construct their own microbiome data analysis pipeline using AWS. The AWS cloud can significantly expedite the microbiome analysis process and provide a collaborative platform for sharing data and results among research collaborators. The MAP-AWS tool successfully completed all microbiome processing and analysis steps both efficiently and reproducibly. The MAP-AWS not only maintains essential reproducibility of processing steps and analyses but also facilitates the efficiency and cost-effectiveness of microbiome data analysis in contrast with basic, commonly used methods of microbiome data analysis [12].

Compared with standard processing for big data analysis, the AWS cloud brings extensive benefits to current microbiome data analysis, including optimized computational capabilities, flexible EC2 instance configurations, and robust security and policies for all resources. Although common server and desktop environments can provide microbiome processing capabilities, AWS brings a supportive systems environment for storage, computational, and analytical capabilities. For instance, many methods in the microbiome platform benefit from compute-optimized processing since their focus is serving high performance computing targeted for compute-intensive applications. The MAP-AWS includes an integrated tool with a combined tutorial for using AWS tools (such as S3 bucket retrieval and EC2 instances use) and performing raw data processing, advanced QIIME 2 and RStudio analysis, and data sharing and management between researchers. This MAP-AWS platform establishes a common environment for sharing analysis tools and results between project managers and researchers across institutions. Given the appropriate permissions, researchers internal to the University and external collaborators can reliably rerun analyses and share findings. It is easy to deploy the microbiome data platform in multiple regions around the world with just a few clicks.

AWS cloud has been widely adopted for whole-genome sequencing (WGS) analysis tasks. For large-scale WGS analyses, AWS was shown to be an efficient and affordable WGS analysis tool [29]. Specifically, Wang and colleagues evaluated the performance of GT-WGS with a 55×WGS data set (400 gigabyte fastq sequences), provided by the genome-wide complex trait analysis (GCTA) 2017 competition, and found that their system took only 18.4 minutes to finish the analysis and that the cost of the whole process was only $16.50 (United States Dollars) [29]. Likewise, our initial microbiome pilot study was completed quickly (within 4 hours) using the MAP-AWS, in contrast with 2-3 days for runs using local computers. Thus, implementing MAP-AWS can significantly improve computing efficiency and speed up the translation of research findings into clinical practice.

Several EC2 pricing models exist, including on-demand, reserved, and spot instances. Users can increase or decrease their computing capacity according to the real-time demand of their applications with on-demand instances and by paying by a specified hourly rate. We tested our microbiome pipeline using on-demand instances, showing a great capacity for microbiome data analysis at a low cost.

One of the biggest challenges facing researchers is the ability to integrate and correlate the massive amounts of data produced by these protocols and identify biologically relevant information that can be used to formulate testable hypotheses. As a proof-of-concept test for the utilization of AWS in microbiome data analysis, our findings support its value and affordability. Our MAP-AWS efficiently integrated and correlated significant amounts of omics data stored and utilized in a cloud-based environment and provided a streamlined platform for communication between researchers. Microbiome research is on the precipice of producing large data sets of great magnitude. To accurately assess and utilize this data, investigators must rely on the development of tools, pipelines, and standard operating procedures to handle big data effectively and efficiently via the AWS cloud. Together, researchers, clinicians, and computer scientists, with the help of AWS cloud computing services, are poised to revolutionize microbiome research and its applications in human health.

Our microbiome data analysis pipeline was undertaken within a cancer nursing research group and tested with data sets of small sample sizes. The technical pipeline should also be applicable to other microbiome data sets, such as oral or skin microbiome data. A dysbiotic human microbiome is associated with a variety of human disease susceptibility [21,30], including endocrine-related disorders (eg, diabetes [31] and inflammatory bowel diseases [32]) and neurodevelopmental disorders (eg, autism spectrum disorders [33] and Alzheimer’s disease [34]). Therefore, the MAP-AWS can be extended to analyze the microbiome data of various chronic diseases and conditions. Our goal is to further test our MAP-AWS using large data sets. In addition, the current pipeline is primarily embedded with QIIME 2 and RStudio, which limits the use of other microbiome analysis packages like Mothur [35]. As QIIME 2 is gaining more attention as a bioinformatics tool, the MAP-AWS is an ideal example of conducting microbiome data analysis with the AWS cloud. As there is increased access to deidentified microbiome data sets, such as the Human Microbiome Project [10], American Gut [11], and the Qiita platform [36], the MAP-AWS will provide our clinical scientists and clinicians a new cloud-based tool to understand the role of the microbiome in quality of care and patient outcomes.

### Conclusions

This study was a proof of concept for building and testing a newly developed pipeline (MAP-AWS) for microbiome data analysis. This pipeline is efficient and highly cost effective. It will provide a convenient environment to share analysis tools and results between collaborators. The long-term goal for this platform is to continue the collaborative use of AWS among clinical scientists across different institutions to make our multidisciplinary research team more productive and insightful. A larger-scale testing of the MAP-AWS across different clinical conditions will enhance communications between multidisciplinary researchers and confirm our proposed efficiencies for running a microbiome pipeline in a cloud-based environment.

## Abbreviations

AWS: Amazon Web Services
CLI: command-line interface
CPU: central processing unit
CRISPR: Clustered Regularly Interspaced Short Palindromic Repeats
EBS: Elastic Block Store
EC2: Elastic Compute Cloud
GCTA: genome-wide complex trait analysis
IRB: Institutional Review Board
IT: information technology
MAP-AWS: microbiome data analysis pipeline using Amazon web services
OS: operating system
QIIME: Quantitative Insights Into Microbial Ecology
RAM: random access memory
RNA-Seq: RNA-Sequencing
S3: Simple Storage Service
SSH: Secure Shell
WGS: whole-genome sequencing
